# Endometriosis in the Lumbar Plexus Mimicking a Nerve Sheath Tumor

**DOI:** 10.4021/wjon413w

**Published:** 2011-12-19

**Authors:** Sunil Jeswani, Doniel Drazin, Ali Shirzadi, Xuemo Fan, J. Patrick Johnson

**Affiliations:** a Department of Neurosurgery, Cedars-Sinai Medical Center, Los Angeles,CA USA; b Department of Pathology, Cedars-Sinai Medical Center, Los Angeles, CA USA; c The Spine Center, Cedars-Sinai Medical Center, Los Angeles, CA USA

**Keywords:** Endometriosis, Nerve root, Synovial cyst, Lumbar radiculopathy, Woman, Cyclical

## Abstract

Endometriosis consists of ectopic endometrial tissue outside of the uterine cavity. It is typically benign. It may cause neurological symptoms if involving the central or peripheral nervous system. We present in this report a 46-year-old Caucasian female with progressively worsening lumbar pain with radiation to her left anterior thigh. MR imaging showed an enhancing mass in the L4 neural foramen, intrepreted as a nerve sheath tumor. At operation the nerve showed extrinsic and intrinsic abnormality, proven to be endometriosis. Postoperatively, the patient reported relief from her radiculopathy. We review the previous cases, discuss the pathogenesis and additional characteristics that highlight intraspinal endometriosis, although rare, should be considered as a potential cause of neurologic symptoms in women. Surgical resection is recommended in cases having severe or worsening neurologic symptoms or signs of cauda equina syndrome. Adjunctive treatment may be used in cases of residual or recurrent lesions.

## Introduction

Endometriosis consists of ectopic endometrial tissue outside of the uterine cavity, frequently occurring in various sites in the pelvis. Endometriosis involving the spinal canal has been previously reported, however, is very rare. Recent evidence indicates that ectopic endometriosis recruits its own unique neural and vascular supply through neuroangiogenesis and influences dorsal root neurons within the central nervous system, thus increasing pain perception in patients [1]. Involvement of the nervous system with endometriosis has been reported in several case reports, in which endometrial tissue has been found involving the sciatic nerve in the retroperitoneal space [2-6]. There have been 6 cases of intraspinal endometriosis described in the literature from the thoracic spine to the conus [7-12].

Treatment of endometriosis in the spine can be challenging. Management options include resection with possible postoperative medical therapy [5, 9]. We present the case of a 46-year-old woman with endometriosis within the neural foramen of L4 mimicking a nerve sheath tumor. Gross total resection was achieved and no postoperative medical therapy was necessary. This case shows that endometriosis, although rare, should be considered in the differential diagnosis of nerve sheath lesions in women.

## Case Report

### History and Examination

This 46-year-old Caucasian female with a past medical history significant for Charcot-Marie-Tooth disease, degenerative arthritis and scoliosis was evaluated for lumbar pain progressively worsening for the last 3 years in her left hip and buttock with radicular symptoms radiating to her left anterior thigh. Her other pertinent history includes a hysterectomy, a left hip replacement, two knee surgeries and an appendectomy in the distant past. Neurological examination demonstrated no focal neurological deficits, but was notable for pain in a left L4 and L5 distribution.

**Figure 1:**
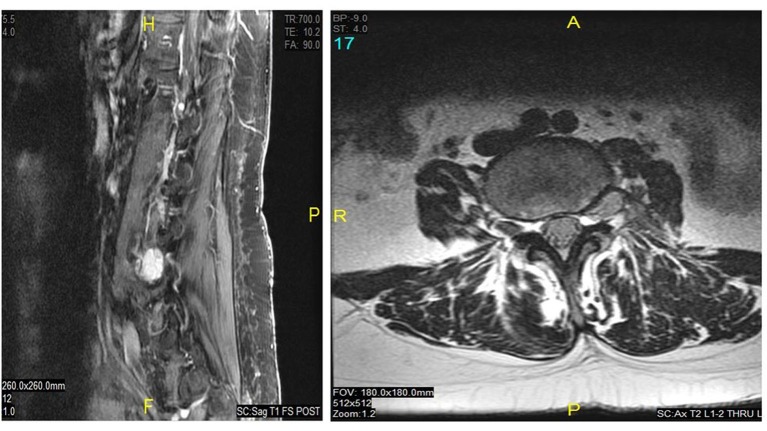
Preoperative T1 sagittal (right) and axial (left) MRI with contrast showed a variable enhancing lesion with possible fluid levels in a multilobulated lesion of the left L4 neural foramen.

A review of imaging studies of the lumbar spine with and without contrast showed a variable enhancing lesion with what appears to be some fluid levels in a multilobulated lesion of the left L4 neural foramen that was read by the radiologist as a hemangioma or possible nerve sheath tumor (Fig. 1). She has had imaging studies as recently as 3 years ago which she reported did not show any abnormality in the lumbar spine.

### Operation

A midline lumbar incision was utilized and intraoperative x-rays were used to confirm the L4-L5 level. Exposure of the intertransverse membrane at the L4-5 level was completed and a left L4-5 hemilaminectomy and removal of the ligamentum flavum was completed. The exiting L4 nerve root was subsequently identified underneath the pars.

Further exposure distally with partial removal of lateral wall of the superior facet joint of L5 was completed to allow visualization of the entire length of the nerve at the L4 level. The nerve appeared to be swollen and enlarged, and appeared to have some fatty components infiltrating within some of the nerve rootlets. Differential dissection around the nerve revealed that there was a soft tissue mass ventrally and extending up to the neural foramen.

Longitudinal incision was made in the nerve. Upon opening the nerve, there was no clear evidence of distinctive intraaxial tumor and there were abnormal appearing nerve fascicles with fatty material in the fascicles. No tumor was identified within the nerve frozen section. The nerve was debulked of all visible tumor, circumferentially, with gentle mobilization. The wound was then assured for hemostasis and irrigated copiously with antibiotic irrigation and self-retaining retractors were removed. At the end of the procedure, the patient was rolled onto the hospital bed, awakened, extubated, and transferred to the recovery room in stable condition.

### Postoperative course

**Figure 2:**
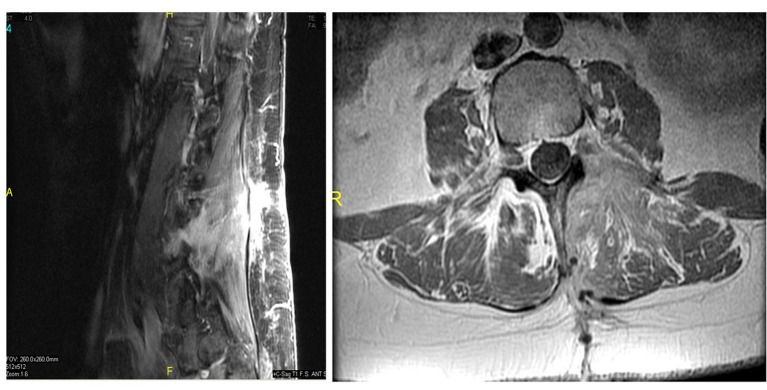
Postoperative T1 sagittal (right) and axial (left) MRI with contrast demonstrating resection of the nerve root sheath tumor.

Postoperatively, the patient reported that she had pain at the incision and intermittent radiation to her left gluteal area, however, she denied any radiation to her left lower extremity. The patient had an uneventful recovery and was discharged home on the 3rd postoperative day. The postoperative MR image revealed resection of the nerve root sheath mass (Fig. 2). She continues to be asymptomatic on follow-up at 18 months.

### Pathology

**Figure 3:**
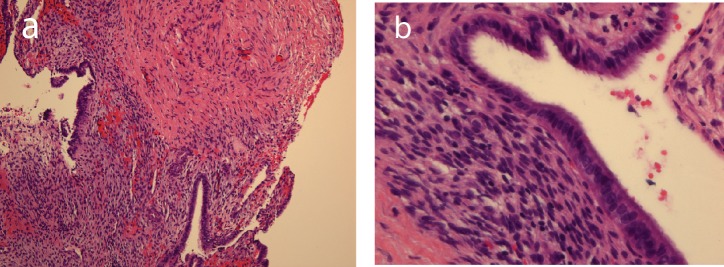
Bland-appearing endometrial glands with associated endometrial stroma and adjacent dense fibrous tissue in medium-power photomicrograph (original magnification, ×10; hematoxylin-eosin stain, a), and in high-power photomicrograph (original magnification, ×40; hematoxylin-eosin stain, b).

Sections showed bland-appearing endometrial glands with associated endometrial stroma with mild early secretory change and adjacent dense fibrous stroma (Fig. 3A and 3B). Focally, there are clusters of hemosiderin-laden macrophages, consistent with remote hemorrhage, however, no apparent cytological atypia is identified.

## Discussion

Endometriosis is a common and benign condition affecting females that is defined by the presence of endometrial tissue in extrauterine locations. Most often, the location of the ectopic tissue is located within the pelvic cavity; however, endometriosis can be found at more distant sites as well. Common symptoms include pelvic pain, dysmennorhea, and infertility. Diagnosis of endometriosis is usually accomplished via direct visual of the lesions through laparoscopic or open approaches. Alternatively, empiric medical therapy can be initiated if the clinical suspicion is high.

### Spinal Endometriosis

Although extrapelvic endometriosis is frequently encountered, involvement causing neurological symptoms is very rare. The clinical presentation, location and treatment of previous spinal endometriosis cases are summarized in Table 1. Symptoms are frequently due to the degree and location of the compression of neural elements from the mass, including pain, weakness, and loss of bowel and bladder control, and may even result in paraplegia. Often times, patients may experience symptoms that fluctuates with her menstrual cycle, however as the mass increases in size the symptoms may become more constant [2-8]. The symptoms may also present if the intraspinal endometriosis becomes hemorrhagic, which is most likely during the menstrual period [7, 11].

**Table 1:** Diagnostic Findings in 7 Patients (Including the Present Case) Reported in the Literature, Including the Clinical Presentation, Location, Treatment and Outcome

Reference, Year	Age	Clinical presentation	Location	Treatment	Adjunctive treatment	Outcome
Agarwal et al, 2006	40	Back pain –menstrual, weakness, incontinence	L1-2, Intradural	Bilateral laminectomy	Danazol, Bilateral oopherectomy	Pain free. No recurrence
Carta et. al., 1992	35	Episodic back and left thigh pain	L3 foramen, Extradural	Bilateral laminectomy, foraminotomy on left side	Recurrence, treated with LH-RH drug	Pain free after retreatment
Erbayraktra et al., 2002	28	Cyclic low back and groin pain, sphincter disturbance	Conus, Intradural	Laminectomy	GnRH therapy postoperatively. Recurrence, treated with repeat laminectomy and oophorectomy	Resolution of sphincter disturbance and back pain
Gortzen et al., 1995	38	Back pain, recurrent monoparesis of left leg, cyclical leg pain	T8-9, only visible during menstruation	GnRH analog as primary treatment	None	Symptom free without neurological deficit
Lombardo et al., 1968	26	Radicular pain, repeated subarachnoid hemorrhages	L1-2 nerve roots, Intradural	Laminectomy	Recurrence, treated with Chlorprogesterone	Occasional pain, no further subarachnoid hemorrhage
Sun et al., 2002	27	Cyclic lumbosacral and radicular pain, leg weakness, dysuria	L3-4, Intradural	Laminectomy	Danazol postoperatively	Pain free, no recurrence
Present case	46	Radicular pain	L4-5 nerve root, Intradural	Hemilaminectomy	None	Pain free, no recurrence

It has been postulated that intraspinal endometriosis probably arises via spread of endometrial tissue via Batson’s venous plexus [7]. Another hypothesis states that embryonic remains of Mullerian structures in retroperitoneum may subsequently develop into endometrial tissue after hormonal stimulation, resulting in endometriosis of retroperitoneal structures such as the spinal canal [1]. Finally, another hypothesis is that the spread of endometrial tissue may occur after surgical operations on the uterus [1, 7]. Review of the literature has shown intraspinal endometriosis to involve the intramedullary, intradural-extramedullary, and extradural compartments, including the vertebral bodies (Table 1).

### Diagnosis

Diagnostic workup may include MR imaging and CSF analysis. Imaging characteristics are often nonspecific demonstrating an intra- or extradural mass. Intraspinal endometriosis may hemorrhage resulting in a hypointense rim on T2 imaging consistent with hemosiderin [7]. The lesion may appear in the intramedullary, intradural, or extradural space. CSF may show signs of prior hemorrhage including xanthochromia and erythrocytes, and moderate protein elevation [7, 8].

### Histology

Histologic analysis remains the gold standard for diagnosis of intraspinal endometriosis, as is the case for endometriosis in general. Histologic analysis will show glandular tissue consistent with endometrium. The histopathology of endometriosis varies with the degree of its response to the normal hormonal fluctuation during the menstrual cycle and the duration of the process. There may frequently be the presence of hemorrhage or hemosiderin, due to the propensity of this tissue to hemorrhage during the menstrual period. Immunochemistry stains will often be positive for progesterone and estrogen receptors. There may also be positivity for tumor associated antigen (CA125) [8]. Immunostain for CD10, ER and PR can be used on rare occasions to support the presence of endometrial stroma.

In a well-sampled specimen, diagnosis is rather straight-forward. However, if specimen is limited or poorly preserved due to hemorrhage, fibrosis, and cystic change or marked cauterized or crush artifact, diagnostic considerations may include hemorrhagic cystic lesion, sarcoma, and metastatic carcinoma. Due to biphasic pattern of endometriosis, synovial sarcoma and carcinosarcoma are also in the differential diagnosis. Malignant transformation is a well-described, rare complication of endometriosis with approximately 75% of these tumors arising from endometriosis of the ovary, although other sites have been described. Endometrioid carcinoma is the most common malignant neoplasm arising within endometriosis, followed by clear cell carcinoma [8].

### Treatment

Treatment options for endometriosis include surgical and medical modalities. During diagnostic laparoscopy, identified lesions may be ablated or excised. Medical therapy includes non-steroidal anti-inflammatory medications, oral contraceptives, GnRH analogues, progestins, and Danazol [5, 9].

Treatment of intraspinal endometriosis may consist of surgical and medical modalities. Surgical resection of intraspinal endometriosis is recommended for patients with severe or worsening neurologic symptoms, or if there are signs of cauda equina syndrome [2, 7, 8]. Complete resection of the endometrial tissue should be attempted; however, this should not proceed at the cost of injuring neural tissue.

Since diagnosis of intraspinal endometriosis is often difficult prior to surgical resection, preoperative drug therapy is often not possible. Postoperatively, the administration of aromatase inhibitor or gonadotrophin releasing hormone analogs such as Danazol has been recommended [2, 6-8]. Additionally, postoperative oophorectomy may also be useful to prevent recurrence [2, 7]. These measures are especially advantageous in those cases where subtotal resection of the intraspinal endometriosis was performed, or in those cases where there was a recurrence.

### Conclusions

Although intraspinal endometriosis is rare, it should be considered as a potential cause of spinal pain or weakness in women. Surgical resection is recommended in cases of patients with severe or worsening neurologic symptoms or signs of cauda equina syndrome but caution must be taken to avoid injury to the neural tissue. Adjuvant medications and/or bilateral oopherectomy may be especially useful in cases with residual or recurrent lesions.
